# Retinal S-opsin dominance in Ansell’s mole-rats (*Fukomys anselli*) is a consequence of naturally low serum thyroxine

**DOI:** 10.1038/s41598-018-22705-y

**Published:** 2018-03-12

**Authors:** Yoshiyuki Henning, Nella Mladěnková, Hynek Burda, Karol Szafranski, Sabine Begall

**Affiliations:** 1 Department of General Zoology, Faculty of Biology, University of Duisburg-Essen, Essen, Germany; 2 Faculty of Science, University of South Bohemia, České Budějovice, Czech Republic; 3 Core Facility Bioinformatics, Leibniz Institute on Aging – Fritz Lipmann Institute, Jena, Germany

## Abstract

Mammals usually possess a majority of medium-wavelength sensitive (M-) and a minority of short-wavelength sensitive (S-) opsins in the retina, enabling dichromatic vision. Unexpectedly, subterranean rodents from the genus *Fukomys* exhibit an S-opsin majority, which is exceptional among mammals, albeit with no apparent adaptive value. Because thyroid hormones (THs) are pivotal for *M-opsin* expression and metabolic rate regulation, we have, for the first time, manipulated TH levels in the Ansell’s mole-rat (*Fukomys anselli*) using osmotic pumps. In Ansell’s mole-rats, the TH thyroxine (T4) is naturally low, likely as an adaptation to the harsh subterranean ecological conditions by keeping resting metabolic rate (RMR) low. We measured gene expression levels in the eye, RMR, and body mass (BM) in TH-treated animals. T4 treatment increased both, *S-* and *M-opsin* expression, albeit *M-opsin* expression at a higher degree. However, this plasticity was only given in animals up to approximately 2.5 years. Mass-specific RMR was not affected following T4 treatment, although BM decreased. Furthermore, the T4 inactivation rate is naturally higher in *F*. *anselli* compared to laboratory rodents. This is the first experimental evidence that the S-opsin majority in Ansell’s mole-rats is a side effect of low T4, which is downregulated to keep RMR low.

## Introduction

### Unusual cone proportion in the mole-rat retina – adaptive or not?

The ability to perceive light was presumably one key advantage in animal evolution explaining the great variability of visual systems found across all animal taxa which have evolved in response to different visual requirements^1^. The mammalian retina possesses two types of photoreceptors, rods for dim light vision and cones for daylight and high acuity vision. Light-sensitive proteins, i.e. opsins, determine the spectral sensitivity of different photoreceptor types. While rods express rhodopsin as the only opsin type, most mammals express two cone opsin types with different spectral properties, namely short-wavelength sensitive (S-) opsins (detecting light in the UV to blue wavelength range, depending on species; encoded by *Opn1sw*, from here: *S-opsin*) and medium-wavelength sensitive (M-) opsins (green wavelength range; encoded by *Opn1mw*, from here: *M-opsin*). Spectral separation of the cone types enables dichromatic vision. Thus far, many exceptions from the mammalian retinal blueprint are known^2,3^, of which one will be addressed in the present study.

African mole-rats from the genus *Fukomys* (formerly known as *Cryptomys*^4^, family Bathyergidae) live in subterranean burrow systems, an ecotope characterised by almost constant darkness. These animals show typical sensory adaptations to the subterranean ecotope^5^, but have retained small, structurally functional eyes. The rod-dominated retina of Ansell’s mole-rats has a high cone proportion of about 10% which is higher than those of nocturnal surface dwellers^6–10^, and can perceive monochromatic light in the blue and green wavelength range^11^. However, while all mammals studied thus far possess a majority of M-opsins in their retina^3,12^, *Fukomys* mole-rats exhibit a clear majority of S-opsins^8^. The retina of the Ansell’s mole-rat (*Fukomys anselli*) contains about 10% pure M-cones, 20% pure S-cones, and 70% dual pigment cones expressing mainly S-opsin and small amounts of M-opsin as determined by immunohistochemistry^8^. Thus, in total, approximately 90% of cones showed a predominant S-opsin expression. The large S-/M-opsin ratio is exceptional in the animal kingdom and has raised questions about a possible adaptive value. However, Kott *et al*.^13^ have shown that blue light propagates less efficiently than green light in natural burrow systems of *F*. *anselli*, and light intensities drop quickly to scotopic levels, by which only rods are activated^13^. These findings make it unlikely that the S-opsin rich retina has any adaptive value for life in subterranean burrow systems. Moreover, brain regions involved in visual orientation in three-dimensional environments are severely reduced in bathyergid mole-rats^14^ explaining recent behavioural data^15^. These findings suggest that the mole-rats’ visual system serves simple light detection rather than high acuity image forming or colour discrimination. Yet, the mechanisms leading to the unique opsin ratio remain unclear.

### Low M-opsin expression might be a side effect of low thyroxine levels

THs, in particular, the transcriptionally active 3,5,3′-triiodothyronine (T3) and the precursor thyroxine (T4), are mainly associated with neural development, growth, metabolism, and the cardiac system, but are also one major factor influencing cone opsin expression^16–21^. Only a small fraction of T4 and T3 secreted by the thyroid gland is circulating freely, while the rest is bound to transport proteins. Only the free fraction (FT4, FT3) is biologically active^22^. In the retina, T3 binds to the TH receptor isoform β2 (TRβ2), a nuclear receptor expressed in cones. This suppresses *S-opsin* expression and instead activates *M-opsin* expression^17,23,24^. Low serum TH levels, a state referred to as hypothyroid, thus leads to downregulation of *M-opsin* expression with a high plasticity throughout adulthood, at least in mice, rats, and likely also in humans^19,25^. Interestingly, we have previously shown that Ansell’s mole-rats have serum FT4 levels which are approximately 10-fold lower compared to other rodents, while FT3 levels are in the rodent-typical range^26^. Total TH levels (free and bound fraction; TT4 and TT3) appeared to be downregulated as well. Moreover, the bound T4 and T3 fraction is significantly higher compared to other rodents, resulting in a lower free fraction relative to total TH levels. These measurements indicate that in *F*. *anselli*, FT4 is downregulated by reduced T4 synthesis and increased protein binding rate. We have interpreted our results in view of the low resting metabolic rate (RMR) of *F*. *anselli*^27^, a typical trait among bathyergid mole-rats^28^. Low RMR is a pivotal adaptation of African mole-rats because in (sealed) underground burrows oxygen availability is low, risk of overheating is high, and food availability is scarce^27,28^. THs regulate metabolic pathways by which the balance between energy expenditure and storage is maintained. Excess THs increase energy expenditure by central and peripheral mechanisms. By the same token, low TH levels reduce energy expenditure^29^. Thus, it appears reasonable that natural selection has favoured low T4 in *F*. *anselli* to maintain low RMR. In contrast, as stated above, there is no reason to assume that a similar selective pressure was acting on colour vision properties. Instead, the S-opsin majority found in *Fukomys* mole-rats is likely to be only a side effect of low T4 levels.

Nevertheless, there is a major contradiction in this model. The normal T3 levels found in Ansell’s mole-rats should be sufficient to upregulate both, RMR and *M-opsin* expression. However, TH signalling is not only dependent on circulating TH levels but also on various other factors. First, THs cannot passively diffuse through cell membranes, thus several transmembrane transporters are required for TH supply of target cells^30^. The monocarboxylate transporter 8 (MCT8) and the organic anion transporting peptide 1c1 (OATP1C1) are well described as high-affinity TH transporters^31^. MCT8 transports T4 as well as T3, with a higher affinity for T3^32,33^, and OATP1C1 has the highest T4 affinity compared to other TH transporters^34^. While MCT8 is widely expressed, including metabolically active organs such as liver and kidney^33^, OATP1C1 is predominantly expressed at blood-tissue barriers^35,36^. Second, intracellular conversion of the prohormone T4 into the receptor-active T3 is catalysed by deiodinases type 1 and 2 (DIO1, DIO2). A third deiodinase type (DIO3) is mostly responsible for TH inactivation into TH metabolites, such as reverse T3 (rT3) or T2^37^. Third, TH action is mediated by binding to TH receptors which are nuclear receptors regulating gene expression. Availability of different receptor isoforms encoded by the genes *Thra* and *Thrb* is cell- and age-specific.^38^. Consequently, availability and function of these regulatory components finally determine cellular TH effects.

In the present study, we treated Ansell’s mole-rats with T4 using subcutaneous osmotic pumps for 12 weeks, to study the effects on opsin expression, RMR, and body mass (BM). We treated another group with T3, to control for T3-specific effects, because T4 treatment leads to a slight upregulation of T3 via deiodination. We aimed to test two scenarios: *i*.*)* if *M-opsin* expression, RMR, or BM are upregulated by T4 treatment, the respective target tissues are likely to be T4-dependent (e.g. through T4-specific transporters), and *ii*.*)* if T4 treatment has no impact on one of these traits, either THs are inactivated in target tissues or the TH signalling pathway is interrupted. By T4 treatment, we successfully achieved rodent-typical TH levels, which allowed us to draw conclusions on these two scenarios, as well as to infer adaptive implications for life underground.

## Results

### T4 administration results in a euthyroid T4 level

**Figure 1:**
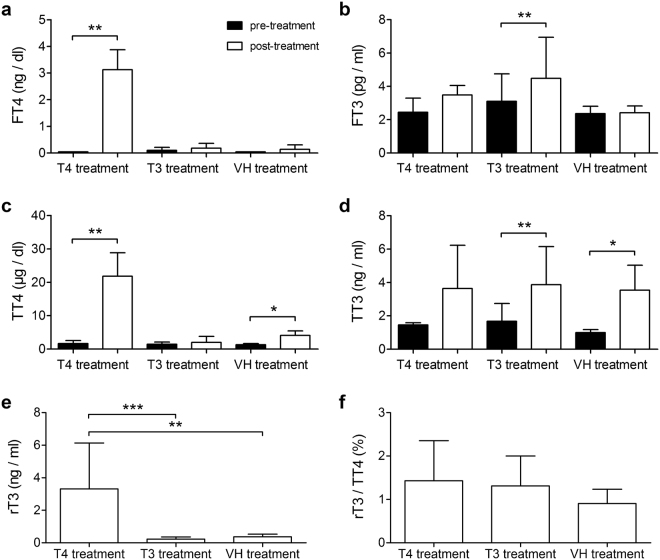
Serum TH levels of Ansell’ mole-rats treated with T4, T3, and VH. Levels of (**a**) FT4, (**b**) FT3, (**c**) TT4, (**d**) TT3, and (**e**) rT3 determined by ELISA. (**f**) The ratio between rT3 and TT4 is plotted separately as a measure of T4 inactivation rate. Pairwise comparisons of log-transformed pre- (baseline, black bars) and post-treatment (treated, white bars) TH levels of the T4, T3, and VH group (**a**–**d**) were done using two-tailed paired t-test. The Benjamini-Hochberg procedure was used to adjust *p*-values for multiple comparisons (threshold FDR 0.05). Intergroup comparison of log-transformed absolute rT3 levels measured in post-treatment samples (**e**) and the log-transformed ratio between rT3 and TT4 (**f**) were done using one-way ANOVA with Bonferroni correction for multiple comparisons. Data are presented as concentrations ($\bar{x}$ ± S.D.) and only adjusted *p*-values are depicted by asterisks. *p < 0.05, **p < 0.01, ***p < 0.001.

We treated Ansell’s mole-rats with T4 (n = 5), T3 (n = 5) or the vehicle solution (VH) (n = 3), in which T3 and VH treatments served as controls. We measured serum levels of FT4, FT3, TT4, and TT3 using commercially available ELISA kits (see Supplementary Table S1), and statistically compared pre- and post-treatment levels to assess successful treatment in each treatment group (only *p-*values adjusted for multiple comparisons are given in this section, unless otherwise stated). In the T4 animals where we aimed to achieve rodent-typical T4 serum levels, FT4 was significantly elevated after treatment (*t* = 40.91, *p = *0.0012) (Fig. 1a). Mean FT4 levels were in the rodent-typical range^26^ (3.12 ± 0.75 ng/dl). FT3 was elevated by a factor of 1.42 (*t* = 2.86, adjusted *p* = 0.0687, unadjusted *p* = 0.046) which can be attributed to T4 deiodination (Fig. 1b). Taken together, T4 supplement resulted in an FT4:FT3 ratio of approximately 9:1, which can be considered rodent-typical^39^. Furthermore, TT4 was also significantly upregulated following T4 treatment (TT4: *t* = 8.87, adjusted *p* = 0.005) (Fig. 1c,d). In the T3 group, we aimed to achieve a slight FT3 upregulation as observed in the T4 group, to control for FT3 effects. Following T3 treatment, FT3 levels were significantly elevated (*t* = 6.53, adjusted *p* = 0.0084) (Fig. 1b) by a factor of 1.45 which is very close to the elevation factor of 1.42 observed in the T4 group. TT3 was also significantly elevated in the T3 group (*t* = 8.39, adjusted *p* = 0.004) (Fig. 1d), but FT4 and TT4 were not affected (Fig. 1a,c). In the VH group, FT4 and FT3 levels did not differ between the two samplings (Fig. 1a,b). Surprisingly, TT4 and TT3 levels were significantly upregulated at the end of the treatment period (TT4: *t* = 10.46, adjusted *p* = 0.022; TT3: *t* = 10.36, adjusted *p* = 0.018) (Fig. 1c,d). Post-treatment levels of rT3, the inactivated form of T4, were compared between the treatment groups, to get an impression of T4 inactivation rates. One-way ANOVA revealed that rT3 was significantly upregulated in the T4 group compared to the other groups (one-way ANOVA, *F* = 20.28, *p* = 0.0003) (Fig. 1e). We further analysed the respective rT3:TT4 ratios (in %), as a measure of relative T4 inactivation rates, but found no significant difference between the three groups (Fig. 1f), indicating that relative conversion rates remain stable despite T4 treatment.

### M-opsin expression is markedly lower expressed than S-opsin on transcriptional level

We measured expression levels of *S-opsin* and *M-opsin* in eyecups containing the retina of untreated Ansell’s mole-rats to determine, if the S-opsin dominance found on protein level^8^ is already set on gene expression level. Real-time Quantitative Reverse Transcription PCR (qRT-PCR) revealed that *S-opsin* was expressed 42 ± 22-fold higher than *M-opsin* as determined by the 2^−ΔΔ*C*T^ method^40^, which indicates that *M-opsin* expression is significantly lower expressed already on transcriptional level (*t* = 24.5, *p* < 0.001, n = 13).

### Opsin expression was upregulated following T4 treatment only in young animals

**Figure 2:**
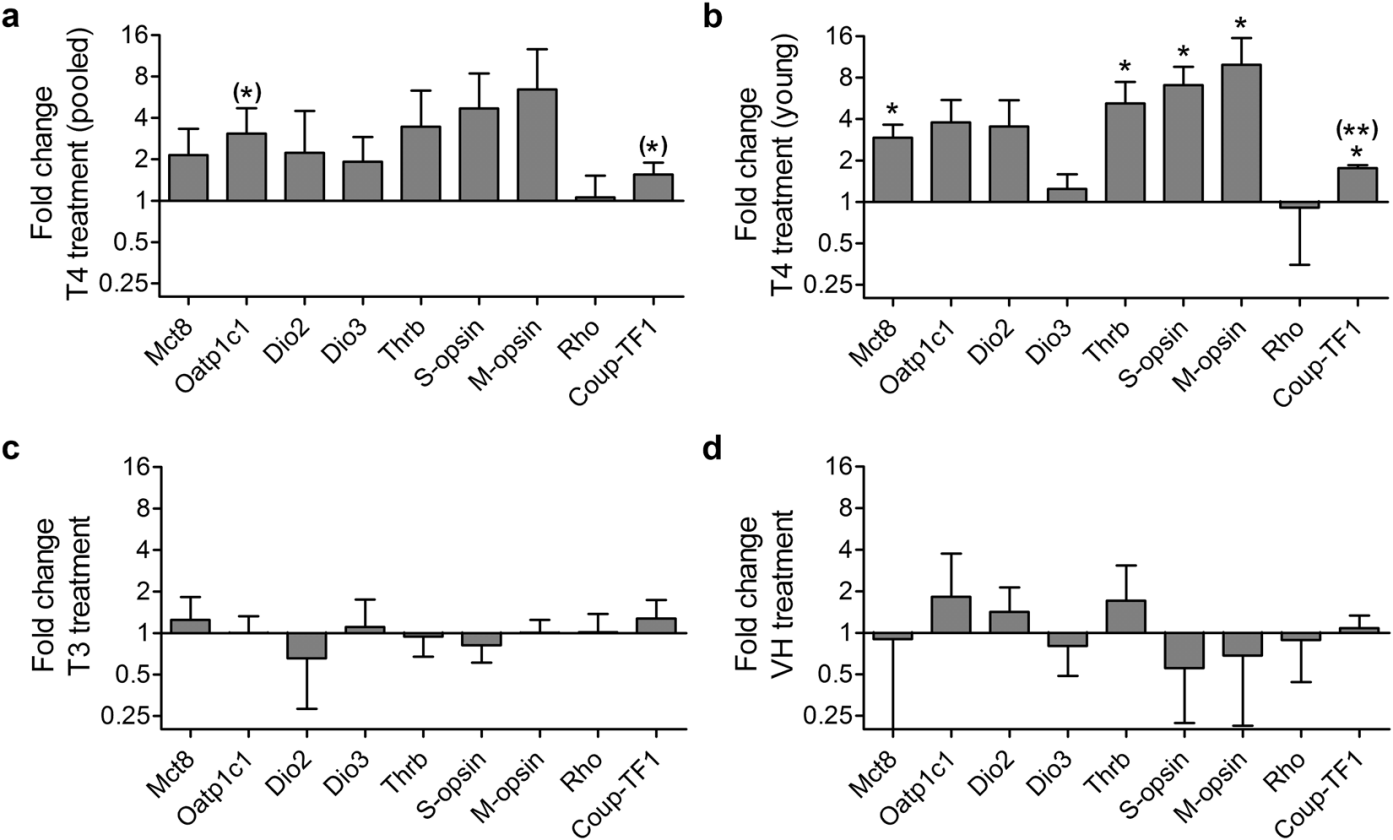
Treatment effects on gene expression levels in eyecup homogenates of *F. anselli*. Relative gene expression levels were determined with qRT-PCR in pre- and post-treatment samples. Samples of the T4 treatment group were analysed as (**a**) pooled data (n = 5) and (**b**) after exclusion of animals >2.7 years (n = 3). (**c**,**d**) T3 (n = 5) and VH treatment (n = 3) had no significant effects on gene expression levels. For statistical analyses (two-tailed paired t-test) data were kept in Ct scale (log_2_) and log-transformed. The Benjamini-Hochberg procedure was used to adjust *p*-values for multiple comparisons (threshold FDR set to 0.05). Unadjusted *p*-values are depicted in brackets, when comparisons were significant before correcting for multiple comparisons. Data are presented as fold changes calculated with the ΔΔCt method between pre- and post-treatment values. Fold changes are presented as $\bar{x}$ ± S.D. *p < 0.05, **p < 0.01.

To test if T4 treatment influenced *M-opsin* expression, we measured gene expression levels of *M-opsin* and *S-opsin* in eyecups containing the retina by qRT-PCR. To determine treatment effects, we extirpated one eye prior to treatment and the other eye post-treatment. By this, we could calculate treatment effects pairwise in each animal (Fig. 2). Relative expression levels of both opsins did not differ significantly between untreated and treated eyes in any of the treatment groups (Fig. 2a,c,d). However, calculation of fold changes revealed a potential difference between young and old animals (boundary arbitrarily set to 1,000 days of age based on fold changes) in the T4 group. While T4 treatment led to an increase of *M-opsin* expression between 6.42–16.34-fold in young animals (n = 3), expression in old animals increased only 1.03 and 1.31-fold (n = 2). Such a differential effect is not seen in the T3 or VH group (see Supplementary Table S2). In a second approach, we therefore analysed the data of young animals only (n = 3). In young animals *M-opsin* expression significantly increased following T4 treatment (Table 1), and also *S-opsin* expression (Table 1;Fig. 2b), indicating that these effects were masked by old animals in the pooled analysis.

**Table 1:** Pairwise analysis of treatment effects on target gene expression in the T4 treatment group. Data were analysed with a two-tailed paired t-test, giving a primary *p*-value and a *p*-value corrected for multiple comparisons (“*p-adj*”) using the Benjamini-Hochberg procedure (threshold FDR 0.05). *Significant treatment effect.

	Pooled (n = 5)			Young (n = 3)		
	*T*	*p*	*p-adj*	*T*	*p*	*p-adj*
*Mct8*	2.08	0.106	0.159	8.31	0.014*	0.032*
*Oatp1c1*	3.94	0.017*	0.153	3.58	0.070	0.105
*Dio2*	0.56	0.605	0.680	0.60	0.610	0.610
*Dio3*	2.31	0.082	0.147	1.33	0.314	0.404
*Thrb*	1.84	0.140	0.180	5.90	0.028*	0.050*
*S-opsin*	2.46	0.070	0.157	8.64	0.013*	0.039*
*M-opsin*	2.68	0.056	0.167	10.37	0.009*	0.041*
Rho	0.16	0.884	0.884	0.65	0.585	0.658
Coup-TF1	3.67	0.022*	0.097	17.66	0.003*	0.029*

**Figure 3:**
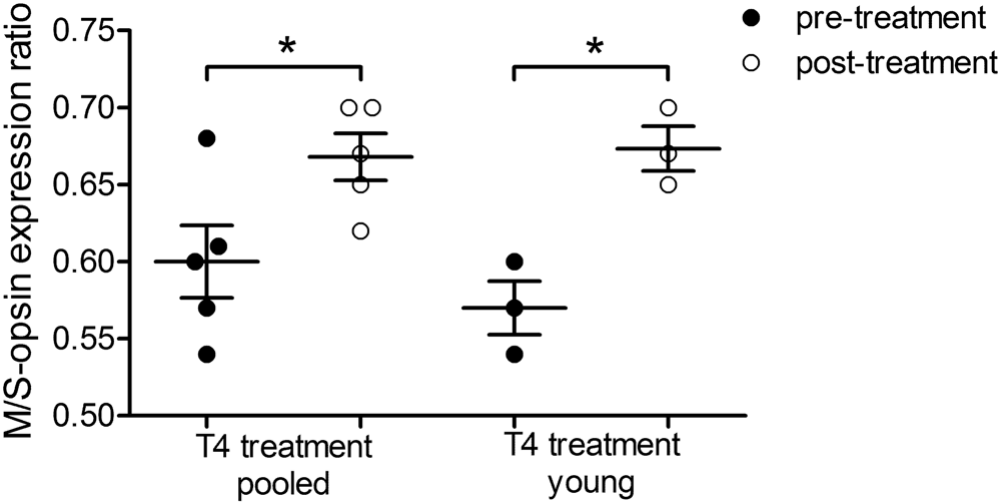
*M-opsin*/*S-opsin* expression ratios pre- and post-treatment in the retina of T4 treated animals. The opsin expression data for each eye were normalised by calculating the *M-opsin*/*S-opsin* expression ratio to control for different baseline expression levels in each eye (pre- and post-treatment). This allows comparison of relative differences between *M*- and *S-opsin* expression between both eyes, an alternative measure for changes in *M-opsin* expression levels. A significant increase was found in pooled data and after exclusion of animals older than 1,000 days (young), indicating that *M-opsin* expression is increased at a higher degree than *S-opsin* expression (two-tailed paired t-test). The effect in pooled data is lower (*p *= 0.046, n = 5) compared to young data (*p *= 0.028, n = 3). Data are presented as median ± interquartile range. *p < 0.05.

To control for the possibility that opsins are expressed at different baseline levels in each eye, we normalised the data by calculating the ratio between *M-* and *S-opsin* expression levels for each eye in pre- and post-treatment samples. By this, we could compare *M-opsin* expression levels in pre- and post-treatment samples relative to *S-opsin* expression as an alternative measure for changes in *M-opsin* expression. An increase of this ratio would further indicate that *M-opsin* expression is upregulated by a higher degree than *S-opsin* and vice versa. We found a significant increase in *M-opsin*/*S-opsin* ratio in the pooled data (*t* = 2.86, *p* = 0.046, n = 5) as well as in data of young animals only (*t* = 5.86, *p* = 0.028, n = 3) (Fig. 3). Here again, the effect in pooled data is lower than in data of young animals. Thus, expression of both opsins is upregulated following T4 treatment, but expression of *M-opsin* is upregulated at a higher degree than *S-opsin* (Fig. 3).

### Expression differences of TH transporters and TH signalling components following T4 treatment

We were further interested if components involved in TH signalling, namely *Mct8*, *Oatp1c1*, *Dio2*, *Dio3*, and *Thrb* are *i*. expressed in eyecup/retina samples, and *ii*. influenced by T4 or T3 treatment. We also measured expression levels of the Rhodopsin-encoding gene (*Rho*) and chicken ovalbumin upstream promoter transcription factor 1 (*Coup-TF1*), a nuclear receptor involved in spatial patterning of cone opsins across the retina^41^. We found that none of the tested components were differentially expressed in the three treatment groups after correction for multiple comparisons (without correction, *Oatp1c1* and *Coup-TF1* were significantly upregulated following T4 treatment; Table 1; Fig. 2a,c,d). In order to test if treatment effects on gene expression are age-dependent, as was the effect on opsin expression, we performed a second, separate analysis in which animals older than 1,000 days were excluded. In young animals, we found a significant upregulation of *Mct8*, *Thrb*, *and Coup-TF1* after correcting for multiple comparisons (Table 1; Fig. 2b). In T3-treated old animals no significant changes in gene expression were found. Unfortunately, we could not statistically analyse treatment effects separately in T4-treated old animals and T3-treated young animals, respectively, because the sample sizes (n = 2) were not sufficient (see Supplementary Table S2).

### Application of T4 decreased body mass but TH treatment did not influence RMR

**Figure 4:**
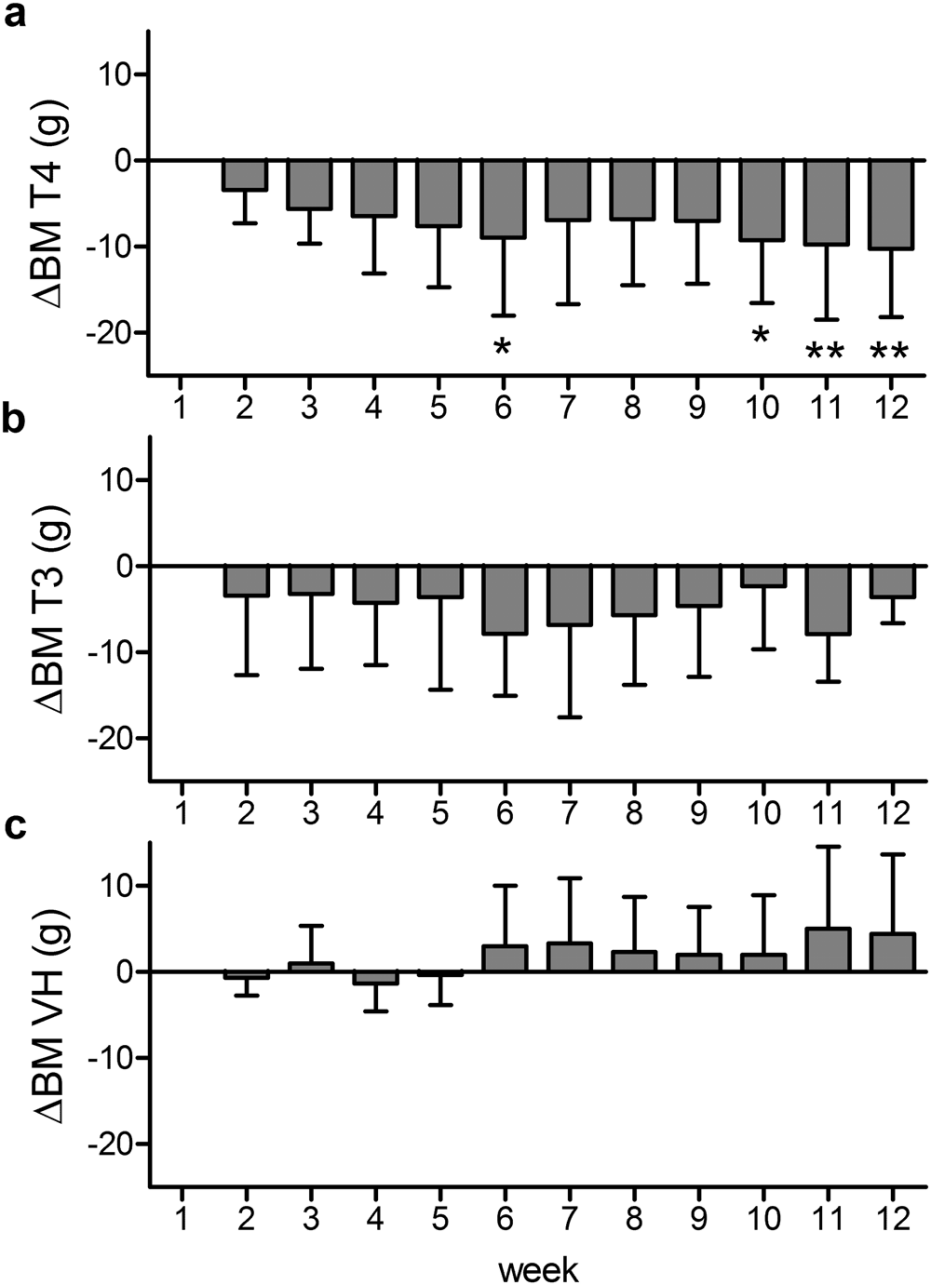
Weekly BM differences to baseline BM (ΔBM) during the treatment period. BMs of the T4 (**a**), T3 (**b**), and VH (**c**) treatment groups were assessed weekly during the 12-week treatment period. Week 1 represents baseline BM (BM at day of pump implantation). Weekly BM was log-transformed and statistically compared for significant differences (repeated measures ANOVA followed by Bonferroni correction for multiple comparisons). BM of T4-treated animals were significantly lower in week 6, 10, 11, and 12 compared to baseline. Data are presented as ΔBM, which was calculated by subtraction of weekly BM from baseline BM ($\bar{x}$ ± S.D.). *p < 0.05, **p < 0.01.

**Figure 5:**
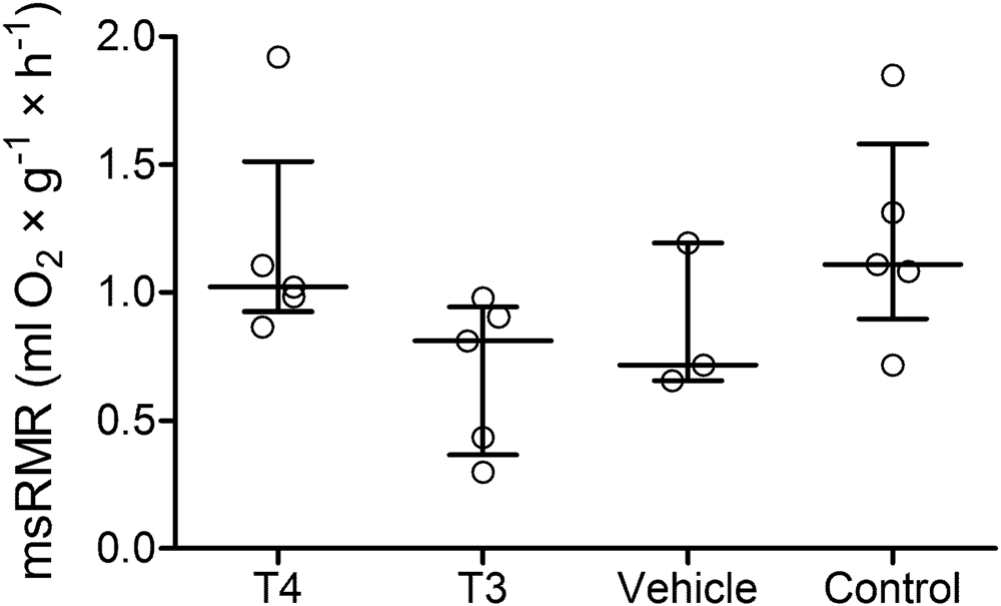
msRMR of the four experimental groups. Resting metabolic rate was measured by indirect calorimetry in all treatment groups with an additional untreated control group and compared with one-way ANOVA followed by Bonferroni correction for multiple comparisons. No significant differences were observed between the groups. Nonlogarithmic values are presented as median ± interquartile range.

At start of the treatments, BM did not differ between treatment groups (one-way ANOVA, *F* = 1.08, *p* = 0.375, n = 3). To reveal possible treatment effects on BM, we calculated a repeated measures one-way ANOVA on weekly BM. In the T4 treatment group, BM differed significantly between week 1 (baseline) and weeks 6, 10, 11, and 12, respectively (*F* = 3.29, *p* = 0.002, n = 12) (Fig. 4). No significant changes in BM were observed in the T3 and VH group. We further measured RMR in all treatment cohorts and an additional untreated control cohort between weeks 10 and 12. No significant differences could be observed in mass-specific RMR (msRMR) between any of the four cohorts (one-way ANOVA, *F* = 2.77, *p* = 0.081, n = 4) (Fig. 5).

## Discussion

In the present study, we investigated how upregulation of T4 impacts retinal gene expression, msRMR, and BM in *F*. *anselli*. The purpose was to investigate the molecular basis of the exceptionally low *M-opsin* expression and to challenge its adaptive viewpoint in a proximate context considering low RMR of mole-rats.

As the basis for the present study, we needed to upregulate serum FT4 levels into a rodent-typical range. A slight upregulation of FT4 by cold acclimation was achieved in naked mole-rats (*Heterocephalus glaber*), a close relative of Ansell’s mole-rats^42^, but this is the first time TH levels were exogenously upregulated under controlled conditions in a mole-rat species. To achieve normal FT4 levels, we treated the animals with a higher T4 dose than recommended by the American Thyroid Association for the treatment of hypothyroid mice^43^, to compensate for the higher protein binding rate in Ansell’s mole-rats^26^. Although total rT3 levels were highly elevated in T4-supplemented animals, the rT3:TT4 ratio, a measure for relative T4 inactivation rate, did not differ between the experimental groups, suggesting that the balance between T4 levels and T4 inactivation stayed equilibrated under T4 treatment. It was instead severalfold higher in all experimental groups (range: 0.91 ± 0.33–1.43 ± 0.93%) compared to rats (0.29%^44^, 0.14%^45^) and mice (0.17%^46^) (rT3:TT4 ratios for rats and mice were calculated with mean values from the cited publications). This indicates that T4 inactivation is naturally upregulated in *F*. *anselli* compared to other rodent species, which might represent one mechanism leading to the naturally low T4 levels. While T4 and T3 treatments yielded the desired results, VH treatment led to an unexpected upregulation of TT4 and TT3. The VH solution contained BSA, i.e. albumin, which is the main serum TH binding protein in rodents^39^ and therefore often used as a stabilising agent to overcome the short half-life of THs during long-term treatments. We assume that artificial administration of BSA could have led to an elevated binding of endogenous T4 and T3 to BSA. This in turn could have led to a drop in free serum TH, which decreases negative feedback to the hypothalamus and pituitary, stimulating endogenous TH release. This explanation is supported by our observation that FT4 and FT3 were unaffected in the VH group, pointing out that only the bound fraction has increased. This finding does not influence our experimental outcomes, since the bound fraction is physiologically inactive, but it should be considered in long-term hormone treatments where BSA is used as a stabiliser.

Advanced short-wavelength sensitivity in Ansell’s mole-rats has no evident adaptive value in underground burrow systems, as suggested by Kott *et al*.^13^ based on light propagation data in burrow systems. In fact, sensory systems of subterranean mammals have adapted to the almost lightless ecotope^5^, thus vision plays a subordinate role. Nevertheless, we were able to upregulate *M-opsin* expression by T4 treatment. This finding reveals that the expression regulation of mole-rat’s *M-opsin* is comparable to the regulation in other mammals, and the S-opsin majority is a systemic effect of low serum T4 levels. However, *M-opsin* expression was only upregulated in young animals up to an age of approximately 2.2 years (fold change: 9.94 ± 5.6; Supplementary Table S2). In older animals (>2.9 years, referred to as old animals), no effect on opsin expression was observed following T4 treatment (fold change: 1.17 ± 0.2; Supplementary Table S2). In the T4 group, we had a gap of approximately 0.7 years between the oldest “young” animal and the youngest “old” animal. Therefore, we could not determine the exact age where *M-opsin* does not respond to T4 treatment anymore, but it is likely to be around 2.5 years. In salmonid fish, TH treatment shows no effect on opsin expression in older animals as well, but this observation is linked to different developmental stages^47^. In *F*. *anselli*, TH sensitivity of cones drops at an age where the animals are fully grown^48^, rendering a developmental cue unlikely. Interestingly, the age-dependent effects we observed in the mole-rat retina are in contrast to Glaschke *et al*.^19^ who, for the first time, showed that *M-opsin* expression is still prone to TH manipulation in fully maturated photoreceptors of adult mice and rats. A similar effect was reported in adult hypothyroid human patients treated with T4, based on a colour contrast sensitivity test^25^. It can be argued that the contradicting observation is a consequence of prolonged natural TH deprivation experienced by the mole-rat retina. The resulting long-term lack of TH signalling might lead to permanent downregulation of redundant components of the TH signalling pathway (e.g. through gene silencing by nuclear orphan receptors^49^), from which *Mct8* and *Thrb* might be promising candidates. Expression of both genes was upregulated in the T4 group, but only in young animals (≤2.2 years). MCT8 plays a role in photoreceptor and interneuron supply with TH, as indicated by immunohistochemical data^50^, and TRβ2 is a transcriptional activator of *M-opsin* expression^16^. Insufficient availability of these two components in old animals might thus impede TH signalling in photoreceptors. But note, the specific role of TRβ2 in mole-rats could not be tested due to insufficient information on the existence and/or structure of the β1 and β2 receptor isoforms. Furthermore, Arbogast *et al*.^51^, who used a reporter mouse line to visualize T3 signalling sites in the retina, have suggested that most T3 signalling is likely to take place in the inner retina^16^. Therefore, expressional changes of *Mct8* and *Thrb* do not automatically reflect changes in T3 signalling within cone cells, but our results revealed promising targets which might be involved in the observed effects on opsin expression.

Unexpectedly, not only *M-opsin*, but also *S-opsin* was upregulated through T4 supplement. This observation is quite puzzling, because in several mammals, *S-opsin* is expressed independently from T3 signalling via TRβ2^16,18,19^. TRβ2 is the isoform responsible for *M-opsin* expression, but it cannot be excluded that T3 initiates other signalling pathways via other receptor isoforms or transcription factors involved in upstream mechanisms. One promising candidate is the retinoic acid receptor-related orphan receptor α (RORα) which transactivates multiple cone genes. RORα also binds to the promoter of *S-opsin* and the locus control region of *M-opsin* and thereby enhances the expression of both genes. RORα-deficient mice do not completely lack opsin expression, but S- and M-opsins are significantly downregulated^52^. Moreover, expression of the RORα gene may be regulated by TH, because expression levels were partly downregulated in brains of hypothyroid mice compared to T4-replaced controls^53^. Taken together, the naturally hypothyroid mole-rat retina might not only lead to low *M-opsin* expression levels, but rather to a general downregulation of both cone opsins (and other cone-specific genes) due to missing RORα signalling. T4 treatment of *F*. *anselli* could thus have augmented expression of both cone opsins via higher RORα availability, while *M-opsin* expression was further enhanced by additional TRβ2 signalling. The latter assumption is further supported by *M-opsin*/*S-opsin* expression ratio, which revealed a significantly higher upregulation of *M-opsin* compared to *S-opsin* expression. We also observed *Coup-TF1* upregulation following T4 supplement. In the retina, the encoded nuclear receptor is pivotal for spatiotemporal patterning of cone opsins^41^. In cultured adipocytes, *Coup-TF1* expression is upregulated by T3, which is in line with our findings^54^. This observation indicates that T4 supplement not only enhances opsin expression in *F*. *anselli*, but might also activate regulatory pathways involved in spatiotemporal patterning. Unfortunately, the sample size available in the present study did not allow us to conduct immunohistochemical studies on retinal flatmounts to resolve this issue on protein level. Note, that in contrast to laboratory mice and rats, mole-rats cannot be purchased from commercial breeders and wild captures cannot be easily imported. The animals have a very slow breeding rate (1–2 litters with on average two pups per litter per year), very low growth rates (0.36 g per day)^48^, and reach full maturity only at >1 year of age. The availability of animals is thus extremely limited, which made us choose qRT-PCR as the method of choice for analysing treatment effects in the eye. This enabled us to screen a broad spectrum of genes for treatment effects and to infer regulatory mechanisms underlying opsin expression. Surely, histological and electrophysiological experiments are needed to support our findings also from functional side, but based on our gene expression data, it can be concluded that the signalling pathways involved in opsin expression are largely inactivated, but can be readily restored by T4 supplement, supporting our main hypothesis of the unusual opsin phenotype in *F*. *anselli* being a side effect of low serum T4. Further support for the hypothesis comes from the RMR measurements. T4 supplement in *F*. *anselli* did not induce changes in msRMR compared to T3 and VH treatment, as well as the untreated control group. This is somewhat surprising, because in mammals, metabolic rate is usually positively correlated with TH serum levels^29,55^. As stated above, T4 inactivation was upregulated following T4 treatment, and overall T4 inactivation rates in *F*. *anselli* are higher than those in the rat. These findings raise the possibility that *F*. *anselli* has evolved tissue-specific resistance to TH signalling. Resistance to TH is usually a pathologic state with different molecular causes, including TH transporters, deiodinases, and TH receptors^56^. However, when mechanisms like elevated TH inactivation are restricted to metabolically active organs such as the liver and kidney, an animal could maintain a low metabolic rate while other TH-dependent tissues are not necessarily influenced. This predicted mechanism could also account for T3 being ineffective in upregulating RMR in mole-rats. Support for this model comes from other animal species, e.g. the blind mole-rat (*Spalax ehrenbergi* superspecies)^57^ inhabiting subterranean burrow systems, and the African striped mouse (*Rhabdomys pumilio*)^58^ inhabiting semi-arid regions with pronounced climatic changes. In both species, a climate-dependent inverse relationship between TH levels and metabolic rate was reported. Thus, organ-specific resistance to TH might represent a shared trait to adapt to challenging environmental conditions. In naked mole-rats, upregulation of FT4 and basal metabolic rate were achieved by prolonged cold exposure (5 °C below normal temperature for >1 year)^42,59^, suggesting that metabolic regulation by T4 is possible in bathyergid mole-rats, when environmental conditions change. In our study, influencing factors such as ambient temperature or food availability were kept constant. Nevertheless, we observed a significant decrease in BM in T4-treated animals, while the T3 and VH group were unaffected. In human, daily T3 administration was shown to upregulate genes related to glucose and lipid metabolism, energy metabolism, and catabolism (among others) in skeletal muscle^60^. If these genes are also upregulated in mole-rats following T4 treatment, this could have led to higher energy expenditure (e.g. through increased activity) and loss of energy stores which might then be causative for weight loss observed in *F*. *anselli*. While the significant drop in BM was established in week 10 (being even more pronounced in week 11 and 12), we also observed a single significant drop in week 6, which we cannot explain thus far. To address these open questions regarding TH effects on metabolic physiology, we are currently planning to investigate the underlying molecular mechanisms in metabolically relevant organs such as liver, kidney, brown adipose tissue, and skeletal muscle in *Fukomys* mole-rats.

In summary, we have, for the first time, manipulated TH levels in a bathyergid species with naturally low T4 serum levels by exogenous TH administration. The finding that T4 treatment led to an upregulation of opsin expression and other regulatory components suggests that retinal TH supply is T4-dependent, supporting previous studies^50,61^. Therefore, the S-opsin majority which was suggested to have no adaptive value in underground burrows^13^, is likely to be a side effect of low T4 serum levels. A low RMR is pivotal to cope with the harsh subterranean conditions, hence we assume that a low T4 serum level is an ecophysiological adaptation to downregulate RMR. Our finding that RMR was the only trait assessed in the present study that was not affected by T4 supplement, supports this assumption. Elevated T4 inactivation is indicative for tissue-specific compensatory mechanisms to keep RMR low. THs regulate animal physiology on many levels, and the present study again emphasizes the need of promoting research in different animal species to understand the full spectrum of TH function.

## Methods

### Animals

We tested a total of 18 Ansell’s mole-rats (8 females, 10 males) which were all born and maintained at the animal facility of the Department of General Zoology, University of Duisburg-Essen, Germany, and hence were of known age (Table 2). The mean age of all animals was 2.6 ± 0.92 years (range: 1.45–4.17 years) at start of treatment, which represents a fully matured adult stage where the prevalence of age-related health impairments are usually not observed in these long-lived rodents with an average lifespan of 7 years and a maximum lifespan of around 20 years^62^. The animals were housed as family groups in glass terraria of appropriate size with wood shavings as litter. Light cycle, room temperature, and humidity were kept constant at 12/12, 25 ± 1 °C and 40 ± 3 %, respectively. The diet consisted of carrots and potatoes fed ad libitum, supplemented with apples and grain provided once per week. All animals remained in their families after completion of the experiments. All animal experiments were conducted in accordance with the German Regulations for Laboratory Animal Science (GV-SOLAS) and were approved by the North Rhine-Westphalia State Environment Agency (LANUV; permit number: 84-02.04.2016.A176).

**Table 2:** Mean age, age distribution, and sex of each treatment group. Age (mean and range) is given in years. The row *Sex* comprises the sample size for each sex in brackets (F = female, M = male). N = total sample size per group.

	T4 treatment	T3 treatment	VH treatment	Untreated
Age, mean	2.29 ± 0.62	2.99 ± 1.3	2.50 ± 0.60	2.93 ± 0.84
Age, range	1.57–2.99	1.45–4.17	1.81–2.88	2.01–3.93
Sex	F (1), M (4)	F (3), M (2)	F (2), M (1)	F (2), M (3)
N	5	5	3	5

### Thyroid hormone treatment

Ansell’s mole-rats were randomly assigned to one of three treatment groups (Table 2) and were treated with a daily dosage of either 90 ng/g BM T4 (T2501, Sigma-Aldrich, Taufkirchen, Germany), 2 ng/g BM T3 (T2877, Sigma-Aldrich) or the vehicle (VH) solution containing 15 mM NaOH, 50% propylenglycol and PBS containing 5% BSA (T844.2, Carl Roth, Karlsruhe, Germany) as monotherapies. Osmotic pumps (pump model 2006, ALZET®, DURECT Corporation, Cupertino, CA, USA) were filled with the respective solution through a 0.22 µm syringe-end filter (Carl Roth, P666.1) and primed in sterile 0.9% NaCl for 60 h at 37 °C before implantation to achieve immediate pumping. Osmotic pumps deliver the test agents with a constant flow rate, thus being well-suited for long-term hormone treatments. Constant administration further overcomes the short half-life of THs in rodents^39^. For implantation, we deeply anesthetized the animals with an intramuscular injection of 6 mg/kg ketamine (Medistar, Ascheberg, Germany) and 2.5 mg/kg xylazine (Medistar)^63^, and implanted the pumps subcutaneously slightly below the scapulae through a small incision. The animals received daily pain medication (Carprofen, 5 mg/kg s.c., Norbrook Laboratories, Newry, UK) for at least 3 days, and were isolated in a sterile terrarium for 24–48 h for recovery before they were returned to their family group. We replaced the implanted pumps after 6 weeks with new pumps.

### Enucleation

Prior to pump implantation, we extirpated the left eye. The right eye was extirpated together with explantation of the second pump after 12 weeks of treatment (end of treatment). By this, pairwise comparisons of treatment effects on gene expression were possible. For enucleation, eyes were lubricated with local anaesthetics (Xylocain, AstraZeneca, Wedel, Germany) to relax the ocular muscles. Afterwards, the eyeball was gently pushed out of the orbit, grasped with a curved forceps at the optic nerve, and pulled out of the orbit by twisting the optic nerve. We immediately removed the cornea, lens, and vitreous from the freshly isolated eyes. The eyecup containing the retina and RPE was transferred into RNA stabilising reagent RNAlater (Qiagen, Hilden, Germany), incubated for 24 h at 4 °C, and stored at −20 °C for expression analyses. Removal of the eyes does not impair the life of mole-rats, because these animals are adapted to the dark subterranean habitat and apart from the capability to distinguish between light and darkness, they do not orient visually^10,14^.

### Quantification of serum TH levels

For monitoring of serum TH levels, we collected blood samples before treatment (baseline), after six weeks of treatment, and at the end of treatments (post-treatment). Blood samples (on average 0.9 ml per sample) were obtained from the vena saphena of the hindpaw under ketamin/xylazine anaesthesia as described above and centrifuged at 10,000 × *g* at 20 °C. Serum aliquots were stored at −20 °C until use. Serum levels of FT4, FT3, TT4, TT3, and rT3 were determined with commercial ELISA kits according to manufacturer’s instructions (DRG Diagnostics, Marburg, Germany: FT4 – EIA 2386; FT3 – EIA 2385; TT4 – EIA 4568; TT3 – EIA 4569; BioVendor, Brno, CZ: rT3 – RCD029R). We measured hormone concentrations of baseline and post-treatment samples, except for rT3 where we only measured post-treatment levels, because baseline serum samples were depleted. In case, FT4 levels were under detection limit of the assay, values are depicted as <0.05 ng/dl, which represents the detection limit of the FT4 ELISA kit used in this study.

### Isolation of RNA and qRT-PCR

Eyecups were immersed in 350 µl lysis buffer (Buffer RLT, Qiagen) and homogenized with a TissueLyser II (Qiagen). Total RNA from eyecup lysates was extracted by using the RNeasy Mini Kit (Qiagen) according to manufacturer’s instructions. An on-column DNase digestion step was further included in the extraction procedure with RNase-Free DNase Set (Qiagen). Total RNA was quantified by using a NanoDrop ND-1000 (Peqlab, Erlangen, Germany). RNA was reverse-transcribed into complementary DNA (cDNA) using AMV Reverse Transcription System (A3500, Promega, Mannheim, Germany) according to manufacturer’s instructions. We further added 10 µg BSA per reaction to reduce the inhibitory effect of melanin on reverse transcriptase and DNA polymerase efficiencies^64^. qRT-PCR was performed using SybrGreen premix (Invitrogen, Carlsbad, CA, USA) in 30 µl final reactions and an iCycler iQ Real Time PCR Detection System (Bio-Rad, Hercules, CA, USA). The PCR program consisted of an initializing step (95 °C for 3 min) and 40 amplification cycles comprising 95 °C for 45 s, 56 °C for 25 s, and 72 °C for 30 s. Each sample/primer combination was measured in triplicate and the Ct values were averaged before normalisation. To obtain normalised mRNA data, we selected two reference genes *Hprt* and *Hexb*, which were shown to be stably expressed in the rodent retina regardless of age and sex^50,65^. Based on the expression levels of the two reference genes, relative expression levels and fold changes of the target genes were calculated following the 2^−ΔΔ*C*T^ method^40^. DNA primer pairs (metabion international, Planegg, Germany) for *Mct8*, *Oatp1c1*, *Dio2*, *Dio3*, *Thrb*, *S-opsin*, *M-opsin*, *Rho*, *Coup-TF1*, *Hprt*, and *Hexb* are listed in Table 3.

**Table 3:** Primers used in gene expression analyses.

Gene	Forward (5′-3′)	Reverse (5′-3′)
*Mct8*	CCTCTAAACCAGGTGCCAGA	GTGAGGTAAGCTGTGCTAGTTGG
*Oatp1c1*	GCTGGTTGTCAAACCTCCAA	ATGCCTCCAAGGGACAAAGT
*Dio2*	GGAAGAGCTTCCTGCTCGAT	GCTGTGACCTCCTTCAGGACT
*Dio3*	GAGCGCCTCTATGTCATCCA	CAGGTGCGAAGCTCTGAGAC
*Thrb*	TGGATGACACGGAAGTAGCC	GCCAGCAGGAAACTATCTTGG
*S-opsin*	ACTGTGGGCACCAAATATCG	ACCTCCCGTTCAGCCTTCT
*M-opsin*	CTGCCCATCCTCACTACTCG	CTGCCGGTTCATGAAGACAT
*Rho*	CATGAGCAACTTCCGCTTTG	GTAGTCAATCCCGCATGAGC
*Coup-TF1*	CATCGTGCTGTTCACGTCAG	TTGGGGTACTGGCTCCTCAC
*Hprt*	TGTTGGCTATGCCCTTGACT	AGGCTTTGTATTTGGCTTTTCC
*Hexb*	TTGGTGGAGAAGCTTGTCTTTG	GTCTCTCGCCAACAGCACTT

### Measurement of resting metabolic rates

RMR were determined by measuring oxygen consumption (VO_2_) during rest by means of an open-flow respirometry system based on our protocol described elsewhere^27^. Briefly, the animals were food deprived for at least 4 h before they were individually placed in a custom-made stainless steel chamber with an airtight acrylic glass (Plexiglas) lid. The chamber was submerged into a temperature-controlled water bath to ensure that all measurements were conducted at 29 °C (within the thermoneutral zone^27^). Each individual was in the respiratory chamber for a minimum duration of 2 h and 3 h maximum. Ambient air was pushed through the chamber with a flow rate of 258 ± 5 ml/min regulated by a flow meter (Model 35830, Analyt-MTC, Müllheim, Germany). Carbon dioxide and water were filtered from the incurrent as well as excurrent air using Soda Lime and Indicating Drierite (Hammond Drierite, Xenia, OH, USA), respectively. Oxygen content of the depleted air was measured by an oxygen sensor (Servomex Type 5200 Multi Purpose, Crowborough, UK) and recorded at intervals of 2 s by DIAdem 8.0 (National Instruments, München, Germany). The lowest 150 consecutive readings (equal to 5 min of measurement) of oxygen consumption were used to calculate RMR following the appropriate equation from Lighton^66^. All measurements were corrected for BM, flow rate, ambient air pressure and temperature.

### Statistical analyses

Statistical analyses were performed using GraphPad Prism 5 (GraphPad Software, Inc., La Jolla, CA, USA). Relative gene expression data were statistically analysed with two-tailed paired t-test^67^ to compare gene expression levels in pre- and post-treatment samples from the same animal. In an alternative approach, we excluded data from old animals (boundary arbitrarily set to 1,000 days of age based on fold changes, see results section for details) and analysed expression data from young animals (n = 3) separately for an age-dependent analysis of T4-treated animals. For analysis, data were kept in Ct scale (log_2_), except for *M-opsin*/*S-opsin* ratios, which were calculated from Ct data prior to analysis. The Benjamini-Hochberg procedure was used to adjust *p-*values for multiple comparisons with the false discovery rate set to 0.05^68^. Figures are presented as fold changes. ELISA data were compared with two-tailed paired t-test for differences between pre- and post-treatment hormone levels, except for rT3 data, which were analysed with one-way ANOVA followed by Bonferroni correction for multiple comparisons to compare intergroup differences at end of treatment. BM data were analysed by comparing weekly BM with repeated measures ANOVA followed by Bonferroni correction for multiple comparisons. Figures are presented as ΔBM, which was calculated by subtraction of weekly BM from baseline BM (baseline equals 0). msRMR data were analysed with one-way ANOVA followed by Bonferroni correction for multiple comparisons. qRT-PCR, ELISA, BM, and msRMR values were log-transformed prior to analysis, but non-logarithmic values are presented in figures. All data are shown as $\bar{x}$ or median ± S.D. or interquartile range, as indicated in figure legends, and significance was defined as *p < 0.05, **p < 0.01, ***p < 0.001.

### Data availability

The datasets generated during and/or analysed during the current study are available from the corresponding author on reasonable request.

## Electronic supplementary material

Supplementary Tables
