# Supplementary material for: Retinal S-opsin dominance in Ansell’s mole-rats (Fukomys anselli) is a consequence of naturally low serum thyroxine
Source: Sci Rep. 2018 Mar 12;8:4337. doi: 10.1038/s41598-018-22705-y (PMC5847620; doi:10.1038/s41598-018-22705-y)
Supplement: Supplementary file 1 — Supplementary Tables [file 41598_2018_22705_MOESM1_ESM.docx]

**Supplementary Information**

**Retinal S-opsin dominance in Ansell’s mole-rats (*Fukomys anselli*) is a consequence of naturally low serum thyroxine**

Yoshiyuki Henning, Nella Mladěnková, Hynek Burda, Karol Szafranski, & Sabine Begall

**Supplementary Table S1: Serum TH levels.** TH levels were determined in pre- (Baseline) and post-treatment (Treatment) serum samples in the three treatment groups (T4, T3, VH) by ELISA. Levels of rT3 were only measured in post-treatment samples. rT3:TT4 represents the ratio between rT3 and TT4 in post-treatment samples. Data are presented as x̅ ± S.D.

|  | T4 treatment | | T3 treatment | | VH treatment | |
| --- | --- | --- | --- | --- | --- | --- |
|  | Baseline | Treatment | Baseline | Treatment | Baseline | Treatment |
| FT4 ng/dl | <0.05 | 3.13 ± 0.75 | 0.10 ± 0.11 | 0.18 ± 0.18 | <0.05 | 0.14 ± 0.16 |
| FT3 pg/ml | 2.45 ± 0.84 | 3.48 ± 0.57 | 3.10 ± 1.65 | 4.48 ± 2.46 | 2.36 ± 0.45 | 2.42 ± 0.41 |
| TT4 µg/dl | 1.68 ± 0.87 | 21.79 ± 7.05 | 1.48 ± 0.65 | 2.00 ± 1.80 | 1.31 ± 0.37 | 4.10 ± 1.37 |
| TT3 ng/ml | 1.45 ± 0.13 | 3.64 ± 2.59 | 1.67 ± 1.07 | 3.87 ± 2.28 | 0.95 ± 0.14 | 2.87 ± 0.85 |
| rT3 ng/ml |  | 3.31 ± 2.82 |  | 0.22 ± 0.14 |  | 0.37 ± 0.16 |
| rT3:TT4 % |  | 1.43 ± 0.93 |  | 1.31 ± 0.69 |  | 0.91 ± 0.33 |

**Supplementary Table S2: Target gene expression levels.** Target gene expression levels determined by qRT-PCR in eyecup samples of the three treatment groups (T4, T3, VH), divided into young animals and old animals (boundary arbitrarily set to 1,000 days of age). Data are presented as Ct values. Ranges are given when n=3.

|  | T4 treatment | | T3 treatment | | VH treatment | |
| --- | --- | --- | --- | --- | --- | --- |
|  | Young (n=3) | Old (n=2) | Young (n=2) | Old (n=3) | Young (n=1) | Old (n=2) |
| *Mct8* | 2.24 – 3.68 | 0.78, 1.15 | 0.96, 0.97 | 0.59 – 1.96 | 0.33 | 0.47, 1.90 |
| *Oatp1c1* | 1.78 – 4.83 | 1.38, 2.63 | 0.97, 1.00 | 0.53 – 1.43 | 0.57 | 0.86, 4.04 |
| *Dio2* | 1.25 – 4.59 | 0.19, 0.38 | 0.34, 1.12 | 0.24 – 0.91 | 1.84 | 0.59, 1.82 |
| *Dio3* | 1.04 – 1.64 | 2.63, 3.24 | 0.77, 1.12 | 0.56 – 2.20 | 0.95 | 0.44, 1.02 |
| *Thrb* | 2.71 – 7.05 | 0.70, 0.95 | 0.69, 1.20 | 0.75 – 1.26 | 1.00 | 0.83, 3.28 |
| *S-opsin* | 4.54 – 9.66 | 0.80, 1.43 | 0.79, 0.97 | 0.62 – 1.07 | 0.17 | 0.74, 0.75 |
| *M-opsin* | 6.42 – 16.34 | 1.03, 1.31 | 0.79, 0.91 | 0.82 – 1.29 | 0.14 | 0.92, 0.99 |
| *Rho* | 0.49 – 1.54 | 1.10, 1.46 | 1.22, 1.54 | 0.68 – 0.87 | 0.69 | 0.57, 1.40 |
| *Coup-TF1* | 1.67 – 1.84 | 0.99, 1.46 | 1.27, 2.02 | 0.74 – 1.23 | 0.89 | 0.98, 1.37 |
